# Monitoring of inhaler use at home with a smartphone video application in a pilot study

**DOI:** 10.1038/s41533-020-00203-x

**Published:** 2020-10-16

**Authors:** Nagesh Dhadge, Madhuragauri Shevade, Nisha Kale, Govinda Narke, Dhananjay Pathak, Monica Barne, Sapna Madas, Sundeep Salvi

**Affiliations:** 1 Central Health Services, Pune, India; 2 Chest Research Foundation, Pune, India

**Keywords:** Outcomes research, Patient education

## Abstract

Inhalation therapy is the basis of the pharmacological management of asthma and COPD. Most patients are trained on the correct use of inhalers by health professionals but after that do patients continue to take them correctly at home remains largely unknown. Video recording of the inhalation technique using a smartphone can be used to evaluate the inhaler technique at home. Through this pilot study, we aimed to understand whether inhaler training given to patients in the outpatient clinic translates into good inhalation practices at home by a video application platform using a smartphone. We recruited 70 newly diagnosed asthma and COPD patients and a pulmonologist trained them to use their inhaler until they were able to use it correctly. Videos of inhaler use were captured by a relative or a friend at home and then sent to an independent reviewer via WhatsApp on Days 1, 7, 14 and 28 (±2). Each step of the inhaler technique was evaluated based on a predetermined checklist with a rating scale of 0 to 10 (10 for all steps done correctly). Out of 70 patients recruited, 30 (42%) sent all videos. We found that, although all patients performed all the steps correctly in the clinic, none of them performed all steps correctly at home even on Day 1 itself of the inhaler use. On Day 1, the steps score reduced from 10 to 6.9 with a downward trend until Day 28. The most common mistakes from Day 1 onwards were incorrect inspiratory flow rates and not gargling after the inhaler use. Also, most patients showed partially effective inhalation as per our scoring method. Remote video monitoring of inhaler use in the home environment is possible with a mobile video application that gives us a better insight into the most common inhaler mistakes performed by patients at home. Inhaler errors start appearing immediately on Day 1 after the training, and incorrect inspiratory flow rates and forgetting to do gargles are common errors. Early detection of inhaler errors at home may be possible through this method.

## Introduction

Inhalation therapy is the cornerstone in the management of asthma and chronic obstructive pulmonary disease (COPD). They produce the therapeutic response at a fraction of the dose used by the oral route, have a quicker onset of action, and lesser side effects than oral medications. Inhalation therapy is mostly delivered by two types of inhaler devices, a pressurized metered-dose inhaler (pMDI) and a dry powder inhaler (DPI). Proper use of each of these devices is key to successful therapeutic outcomes. The physician, nurse assistant, or pharmacist often teaches the correct use of inhaler devices to their patients, although the quality of teaching varies from place to place and from individual to individual. How much of this training the patient retains is not known but is often determined by the quality of training, inhaler type, age, gender, education status, and socioeconomic status^1,2^. After receiving inhaler technique training, unsupervised correct inhaler use at home by the patient is most likely to have a major influence on symptom scores and quality of life of asthma or COPD patients. However, after receiving training in the outpatient clinic, little is known about how in reality patients take their inhalers at home or what errors do they make in their home environment.

It is well known that the amount of drug deposition of inhaled aerosols is the most critical aspect of inhaler technique for achieving a maximal therapeutic effect in the treatment of obstructive airway disorders^3,4^. This can only be achieved by an error-free inhaler technique by the patient at home or in a workplace environment. Errors in the inhalation technique are strongly linked to poorer outcomes, reduced therapeutic efficacy, increased cost, and the use of healthcare facilities^5,6^. Although there have been extensive improvements in the design of various inhalers that yield better drug deposition, inhaler errors still remain disappointingly high in real life^7–9^.

To minimize errors during the use of inhaler devices at home, retention of the correct inhaler technique by the patient as demonstrated in a pulmonologist’s office is a pre-requisite for good execution. But many studies show poor retention and subsequent deterioration of the memory of inhaler training learned over a period of time^10–14^.

This makes monitoring of inhaler technique at home and, if required, subsequent correction of the inhaler technique an important component in the treatment of asthma and COPD^15,16^. To achieve the highest possible inhaler competence in the inhaler use, there is a pressing need to aggressively monitor inhaler usage by a simple and cost-effective method. Moreover, it will be useful to monitor patient’s inhaler technique in their home environment, which ultimately influences disease symptom scores, as clinic-based evaluation of errors may not be entirely reflective of inhaler use in the home environment^11^. At present, in clinical practice, the inhaler technique is evaluated periodically only on follow-up visits in the clinic usually after a gap of few weeks^17^. Despite teaching the correct use of inhaler devices, many patients forget certain steps reducing the efficacy of the inhaled medications. Very few studies have evaluated the correctness of the use of inhaler devices at home.

There is a smartphone boom in India, where there are more users in India than any other country in the world^18^. According to the National Institution for Transforming India Aayog, it is estimated that there are about 400 million Indians with a smartphone who use WhatsApp^18^. Cameras in a smartphone can record photos and videos, which are shared easily and freely with friends and relatives. By taking the help of the rapid growth of mobile technologies in healthcare management, a smartphone and video messaging applications like WhatsApp can be used to monitor the use of inhalers in the home environment^19^. Subsequently, physicians then can intervene in case of errors as quick feedback is known to improve the inhaler technique^20,21^. WhatsApp is allowed for the use of telemedicine in India (https://www.mohfw.gov.in/pdf/Telemedicine.pdf, access date 23-06-2020). Through this pilot study, we assessed the feasibility to use a digital mobile platform to monitor our patient’s inhaler use in their home environment using the WhatsApp messaging application.

## Results

### Demographics and clinical characteristics

**Fig. 1:**
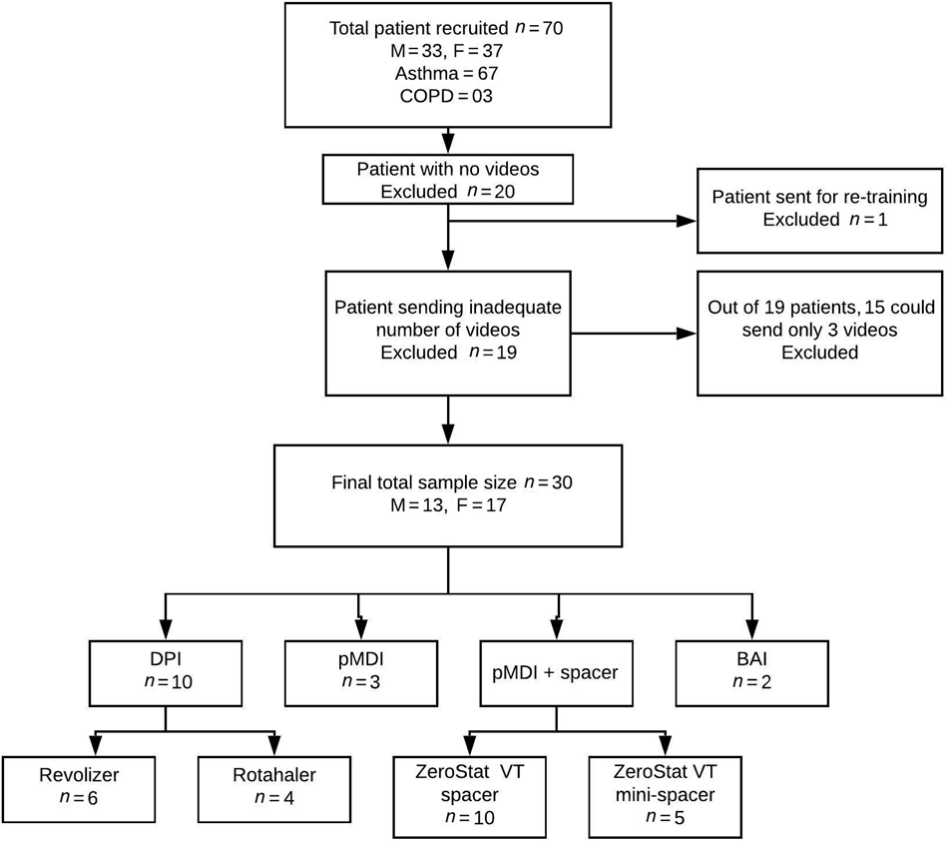
Inhaler devices used in the study. M male, F female, DPI dry powder inhaler, pMDI pressurized metered dose inhaler, BAI breath actuated inhaler.

Seventy patients (33 males, 37 females) between the age range of 18 and 60 (mean 45.5 ± 11.3) years consented and agreed to participate in the study (Fig. 1). There were 67 asthma and 3 COPD patients.

### Data collection

Among these, 20 (28%) patients did not send even a single video. Nineteen (27%) sent videos on a few days. Out of these 19 patients,15 (21%) patients sent only 3 videos, which were just short of 1 video required for inclusion in the study. One patient made >70% mistakes on Day 1 and hence was excluded from the study. Thirty (42%) patients sent all videos on all 4 days as required for the study duration making the final sample size of 30 (13 males and 17 females, 10 DPIs, 20 pMDIs) after all exclusions.

### Inhaler step score

**Fig. 2:**
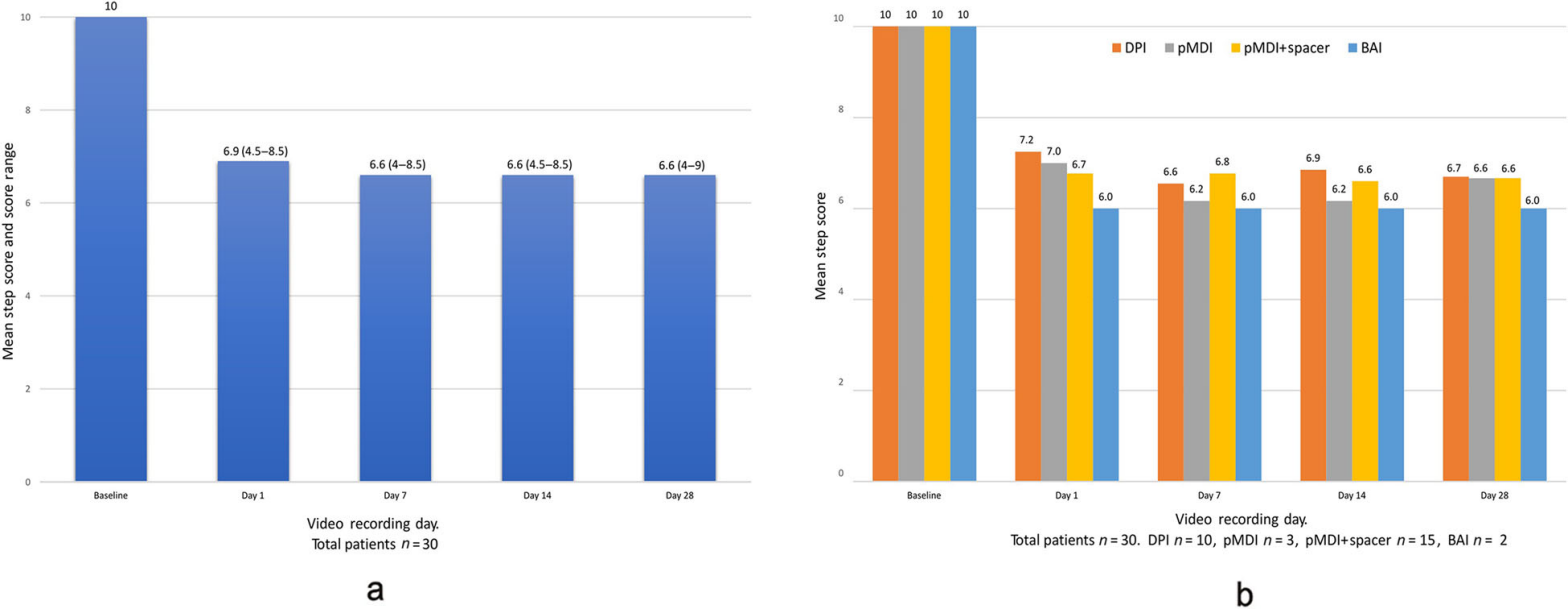
Mean step score with range. **a** All devices. **b** Steps performed correctly per device. Range for DPI (Day 1 video 6.5–8, Day 7 video 5–8.5, Day 14 video 5.5–8, Day 28 video 4–8). Range for pMDI (Day 1 video 6–8, Day 7 video 4.5–8, Day 14 video 4.5–8, Day 28 video 6–8). Range for pMDI + spacer (Day 1 video 5–8.5, Day 7 video 4–8.5, Day 14 video 5–8.5, Day 28 video 4–9). Range BAI (Day 1 video 5–7, Day 7 video 4–8, Day 14 video 5–7, Day 28 video 5–7).

Overall, the quality of videos sent was satisfactory to score all the steps of inhaler use. On Day 1, irrespective of the device used the steps score reduced from 10 on the training day (Day 0) to 6.9 (95% confidence interval (CI) 6.5–7.2) on Day 1 with a declining trend to a score of 6.6 (95% CI 6.1–7) on Day 28 (Fig. 2a). Video recording showed steps score of 6 (95% CI 5.5–6.5) from Day 1 to Day 28 for breath actuated inhaler (BAI), from 6.7 (95% CI 6.3–7.1) to 6.6 (95% CI 6.1–7.1) for pMDI with spacer, from 7 (95% CI 6.6–7.3) to 6.6 (95% CI 6.2–7) for pMDI, and from 7.2 (95% CI 6.9–7.5) to 6.7 (95% CI 6.2–7.1) for DPI. The steps score for all devices was <7.5 throughout the study period (Fig. 2b). While comparing correct steps performed across all the devices, the DPI group performed relatively better on Day 1 than other devices, but the score kept reducing until Day 28 while the BAI group had the lowest steps score of 6.

### Inhaler error rate

While assessing inhaler errors done in each device group over 4 weeks, in the DPI group (*n* = 10) we found 50–70% of the patients doing the important step of quick and deep inhalation incorrectly and 80% not gargling after the inhaler use (Table 1a). In the pMDI group (*n* = 3), all patients failed in the important step of inhalation coordination while 33–67% failed in the breath-hold step (Table 1b). Exhaling through the nose after breath-hold and gargling with water were next common mistakes ranging from 0 to 33% and from 0 to 66%, respectively. In the pMDI with spacer group (*n* = 15), 73–87% erred in the correct time of inhaler actuation, and 40–80% in breathing out before inhalation. 67–80% forgot gargling after inhaler use (Table 1c). In the BAI group (*n* = 2), 50% erred in slow and deep inhalation, 50–100% in gargling and 100% showed error in the step of exhaling through the nose after breath-hold. This was followed by mistakes in the step of shaking and opening the inhaler at 0–50%.

**Table 1:** (a) Most common errors of DPI. (b) Most common errors of pMDI. (c) Most common errors in pMDI + spacer. (d) Most common errors in BAI.

Steps	Day 1 video	Day 07 video	Day 14 video	Day 28 video
(a) DPI (*n* = 10)				
Holds device and inserts capsule	(*n* = 0) 0%	(*n* = 1) 10%	(*n* = 0) 0%	(*n* = 1) 10%
Twists/closes device	(*n* = 0) 0%	(*n* = 1) 10%	(*n* = 0) 0%	(*n* = 1) 10%
Gentle deep exhalation	(*n* = 3) 30%	(*n* = 6) 60%	(*n* = 5) 50%	(*n* = 5) 50%
Mouthpiece between teeth and seals	(*n* = 1) 10%	(*n* = 1) 10%	(*n* = 1) 10%	(*n* = 1) 10%
Inhales quickly and deeply	(*n* = 5) 50%	(*n* = 7) 70%	(*n* = 7) 70%	(*n* = 7) 70%
10 s breath-hold	(*n* = 0) 0%	(*n* = 2) 20%	(*n* = 2) 20%	(*n* = 0) 0%
Exhales through nose	(*n* = 4) 40%	(*n* = 5) 50%	(*n* = 5) 50%	(*n* = 5) 50%
Gargles	(*n* = 8) 80%	(*n* = 8) 80%	(*n* = 8) 80%	(*n* = 8) 80%
Error realization	(*n* = 10) 100%	(*n* = 10) 100%	(*n* = 10) 100%	(*n* = 10) 100%
(b) pMDI (*n* = 3)				
Cap open and shakes inhaler	(*n* = 0) 0%	(*n* = 0) 0%	(*n* = 0) 0%	(*n* = 0) 0%
Gentle deep exhalation	(*n* = 0) 0%	(*n* = 0) 0%	(*n* = 0) 0%	(*n* = 0) 0%
Mouthpiece between teeth and seals	(*n* = 0) 0%	(*n* = 0) 0%	(*n* = 1) 33%	(*n* = 0) 0%
Actuation and slow deep inhalation	(*n* = 3) 100%	(*n* = 3) 100%	(*n* = 3) 100%	(*n* = 3) 100%
10 s breath-hold	(*n* = 1) 33%	(*n* = 2) 67%	(*n* = 2) 67%	(*n* = 2) 67%
Exhales through nose	(*n* = 0) 0%	(*n* = 1) 33%	(*n* = 1) 33%	(*n* = 0) 0%
Gargles	(*n* = 0) 0%	(*n* = 2) 67%	(*n* = 2) 67%	(*n* = 2) 67%
Error realization	(*n* = 3) 100%	(*n* = 3) 100%	(*n* = 3) 100%	(*n* = 3) 100%
(c) pMDI with spacer (*n* = 15)				
Open cap and shake inhaler	(*n* = 3) 20%	(*n* = 4) 27%	(*n* = 4) 27%	(*n* = 1) 7%
Assemble spacer	(*n* = 0) 0%	(*n* = 1) 7%	(*n* = 0) 0%	(*n* = 1) 7%
Attach correctly to the spacer	(*n* = 0) 0%	(*n* = 1) 7%	(*n* = 1) 7%	(*n* = 0) 0%
Gentle deep exhalation	(*n* = 7) 47%	(*n* = 6) 40%	(*n* = 12) 80%	(*n* = 9) 60%
Hold mouthpiece between teeth and seal lips	(*n* = 1) 7%	(*n* = 1) 7%	(*n* = 1) 7%	(*n* = 0) 0%
Actuation and slow deep inhalation	(*n* = 13) 87%	(*n* = 11) 73%	(*n* = 11) 73%	(*n* = 13) 87%
10 s breath-hold	(*n* = 0) 0%	(*n* = 4) 27%	(*n* = 3) 20%	(*n* = 14) 40%
Exhales through nose	(*n* = 7) 47%	(*n* = 5) 33%	(*n* = 5) 33%	(*n* = 4) 27%
Gargles	(*n* = 12) 80%	(*n* = 12) 80%	(*n* = 12) 80%	(*n* = 10) 67%
Error realization	(*n* = 15) 100%	(*n* = 15) 100%	(*n* = 15) 100%	(*n* = 15) 100%
(d) BAI (*n* = 2)				
Cap open and shakes inhaler	(*n* = 1) 50.00%	(*n* = 0) 0.00%	(*n* = 1) 50.00%	(*n* = 1) 50.00%
Gentle deep exhalation	(*n* = 0) 0.00%	(*n* = 0) 0.00%	(*n* = 0) 0.00%	(*n* = 0) 0.00%
Mouthpiece between teeth and seals	(*n* = 0) 0.00%	(*n* = 0) 0.00%	(*n* = 0) 0.00%	(*n* = 0) 0.00%
Slow deep inhalation	(*n* = 1) 50.00%	(*n* = 1) 50.00%	(*n* = 1) 50.00%	(*n* = 1) 50.00%
10 s breath-hold	(*n* = 0) 0.00%	(*n* = 1) 50.00%	(*n* = 0) 0.00%	(*n* = 0) 0.00%
Exhales through nose	(*n* = 2) 100.00%	(*n* = 2) 100.00%	(*n* = 2) 100.00%	(*n* = 2) 100.00%
Gargles	(*n* = 2) 100.00%	(*n* = 1) 50.00%	(*n* = 2) 100.00%	(*n* = 2) 100.00%
Error realization	(*n* = 2) 100.00%	(*n* = 2) 100.00%	(*n* = 2) 100.00%	(*n* = 2) 100.00%

### Inhaler technique efficiency

As per our method of calculating the effectiveness of inhalation, most patients, 93–100%, showed partially effective inhalation throughout the monitoring period of 4 weeks. Strikingly, none of them showed 100% effective inhalation (Table 2). Very few patients (7%) had ineffective inhalation on Days 7 and 28 of the monitoring.

**Table 2:** Inhalation effectiveness score.

Effectiveness score	Day 1 video	Day 7 video	Day 14 video	Day 28 video
	(*n*) Percentages	(*n*) Percentages	(*n*) Percentages	(*n*) Percentages
Ineffective inhalation (%) (≤4)	(*n* = 0) 0%	(*n* = 2) 7%	(*n* = 0) 0%	(*n* = 2) 7%
Partially effective inhalation (%) (4.1–9)	(*n* = 30) 100%	(*n* = 28) 93%	(*n* = 30) 100%	(*n* = 28) 93%
Effective (%) (9.1–10)	(*n* = 0) 0%	(*n* = 0) 0%	(*n* = 0) 0%	(*n* = 0) 0%

We also observed interesting inhaler errors done by some of the patients during video monitoring (Table 3). No patient realized their mistakes while taking inhalers at home or was found correcting them in all the groups during the study period.

**Table 3:** Other observations during video monitoring.

● The patient with pMDI actuated the inhaler correctly but inhaled through the nose instead of the mouth. All the medicine escaped from the device
● The patient with pMDI and spacer actuated the inhaler in air and then attached it to the spacer for inhalation
● The patient closed mouth during breath-hold but continued breathing through the nose
● Patient was drinking water instead of gargling
● Actuating pMDI multiple times in spacer before inhaling

## Discussion

Inhalation therapy is the safest, fastest, and most effective route of drug delivery to the lungs, yet the inhaler devices are very poorly used in clinical practice. Further, inhaler monitoring is the key to the successful management of asthma and COPD patients. For the same reasons, in-person inhaler technique check at the follow-up visit is emphasized in various practice guidelines as inhaler errors are linked to suboptimal symptom control^12,13,15,16,22,23^. But alternative means and methods of monitoring are not laid down in the guidelines. In this context, monitoring of inhaler use at home and its usefulness in clinical practice is not well studied in real-life clinical settings.

In the past, monitoring of inhaler use by video recording in the clinic has been attempted^24,25^. Recently in a small study, video monitoring using a mobile device platform (mobile direct observation of therapy) was found to be useful and acceptable in children for remote monitoring, which also tested corrective intervention^26^. Similarly, a web-based store and forward telehealth system was used for asthma management—a relatively complex system for monitoring than our method^27^. In contrast, we utilized freely available messaging application—WhatsApp for video monitoring, which does not always require training for use.

To the best of our knowledge, this is the first study that has evaluated the use of a commonly used smartphone video application “WhatsApp” for the correctness of the use of inhaler devices (both DPI and pMDI) in a home setting in patients with asthma and COPD. This study gave us an important insight into how patients use their inhaler devices at home after being appropriately trained by the physician in the clinic. Although this was a pilot study that studied only 30 patients out of a total of 70 enrolled, it offers an innovative and simple method of monitoring the use of inhaler devices at home for patients with asthma and COPD that can be scaled up easily without much additional cost. This, in turn, has the potential to have a significant impact on the quality of care that can be offered to patients of asthma and COPD thereby reducing suffering, deaths, and improving quality of life.

The important observation in our study was that inhaler errors were seen from the very first day post-training after performing all the steps well in the clinic. Many of the patients fell into a partially effective inhalation category as per our scoring method, and none of the patients realized inhaler errors on their own so that they could correct their inhaler technique in the home environment. Although all investigators (total three) at two study sites followed the same standard protocol to educate all patients on inhaler technique, there is a possibility that the ability of newly diagnosed patients to absorb this training fully in the first few weeks and quality of training may have impacted their performance. Previously some studies rechecked the inhaler technique in old cases of COPD or asthma on follow-up in the clinic after a few months^5,28,29^. In the study by Gregoriano et al., incorrect inhaler technique ranged from 0 to 53% on follow-up clinic visit after an interval of 2 months. They observed a 20% error in inhalation coordination in pMDI users followed by a 17% error in deep and slow inhalation^30^. In another study by van der Palen et al. in-person check of inhaler technique in the clinic, the mean step score was 72.7 (in percentage) in COPD patients with an average disease duration of 5 years. As in our study, they observed that DPI patients performed better than pMDI in the technique^28^. Comparatively lower steps score and higher inhaler errors in our study could be due to the selection of newly diagnosed patients who never had any previous experience of inhaler use and the fact that inhaler monitoring started very early (starting Day 1 post-training). These differences and quality of training may also have contributed to higher error rates in the inhaler technique found in our study. But our method of inhaler training in the study closely resembles routine clinical practice in asthma and COPD patients.

These observations in the study shake our generally held belief that deterioration in inhaler training memory may take a few weeks or months and may not be so early as Day 1 post-training^10^. This rapid deterioration in the memory of inhaler training in the home environment could point toward the challenges patients face in the home environment. Considering the results of the study, it will be useful to monitor in the home environment immediately after training and preferably in real time for early detection of inhaler errors thus creating an opportunity for corrective interventions to be applied much earlier. Our method does not incur any extra cost for software or training of patients as done for methods used in earlier studies^26,27^.

However, many patients could not complete the study. There could be several possible reasons for this, such as patients discontinuing inhaler therapy as all were newly diagnosed patients, unwillingness to share videos later on despite explaining the study and purpose, feeling shy, patient traveling, emergency personal engagements clashing with sending videos, forgetfulness, emergency medical admission, and technical difficulties in sending videos. Fifteen patients were just short of sending one video out of a total of four required excluding them from the study. More flexibility in sending video might have improved the sample size. If it was not for this pilot study, in real clinical practice even three videos may have helped in checking the inhaler technique. We utilized only one rater to score the inhaler steps in this pilot study because our focus was on understanding feasibility of remote video monitoring in a home environment and other concern was privacy in sharing data with the second evaluator. If privacy concerned are addressed, then using two evaluators for assessment will achieve better accuracy of steps scores and error rates of inhaler technique as the interrater agreement is found to vary significantly for some of the inhaler steps^31^.

Smartphone users in India and worldwide were expected to reach 2.7 billion by 2019^32^. Mobile technologies figure prominently in healthcare services for diagnosis, health education, monitoring compliance with treatment, etc^33,34^. So it is likely that in future more application-based healthcare technologies will be employed in reducing the burden of monitoring of treatment by healthcare professionals. WhatsApp is a secured platform with end-to-end data encryption and facility to communicate through video messages. Its use by young adults was found to be acceptable for asthma care in Latin America^35^. Also, improvement in the compliance and monitoring of the treatment through video using a smartphone has been shown to be beneficial^26^. The awareness of being remotely monitored by video may have a positive impact on inhaler competence as the patient realizes that the focus is on the correct inhaler technique in the treatment^36^. Besides scoring steps of inhaler use using video recording has a good interobserver agreement^24,37^.

WhatsApp is a good, low-cost alternative for use in low-income or developing countries. However, in developed countries, this may be a challenge due to concerns about data privacy. In the future, specific applications for smartphones may be developed that includes enough security.

Remote video monitoring through WhatsApp using a smartphone appears to be a feasible new method of inhaler monitoring in asthma and COPD with an advantage of early detection of inhaler errors. Based on the results of our study, a larger study can be planned to further support our findings.

There were some limitations to our study. First, we had a small sample size due to the high dropout rate. Second, as applicable to any other digital health data, security, confidentiality, and privacy of inhaler videos may be of some concern during inhaler technique monitoring by this method. However, employing safety measures can make this method reasonably safe. Several security features can be utilized such as locking the device with a password or fingerprint lock, activation of device location feature to remotely erase data in case of loss of the device, using antivirus software, and disabling Bluetooth features. Once steps in the inhaler technique are scored, videos can be deleted. The mobile device can be stored in a secured place in the clinic to prevent unauthorized access. Third, only one independent evaluator scored the inhaler technique instead of two, which may have influenced the scoring of the inhaler technique. Finally, WhatsApp use for remote monitoring of inhaler technique will be subjected to the local regulations and guidelines in healthcare services and at present may not be allowed in some countries for monitoring.

## Methods

### Study patients

We recruited 70 physician-diagnosed, new cases of asthma or COPD patients aged between 18 and 60 years from two respiratory clinics in the Pune city.

### Study design

After the diagnosis of asthma or COPD was made, the physician explained the diagnosis and treatment plan to the patient. An inhaler device was selected based on the ability of the patient to use the selected device correctly. The patient was then trained to use the device by the pulmonologist until they were able to perform all the steps correctly. This was evaluated by scoring each step to a total of 10 points per device. Once the patient received a score of 10, the patient and their family member were trained to video record the use of inhaler device by the patient. They were given instructions on how to send recorded videos to the evaluator using the WhatsApp application from their mobile. All investigators followed the same protocol (Annexure I) for the training of various inhaler devices used in the treatment ensuring the same level of training of patients. Our study included two-unit dose DPIs—Revolizer® and Rotahaler®, pMDI without a spacer, pMDI with a spacer, pMDI with Minispacer, and a BAI. A standardized score sheet of the steps of the inhaler technique for each device was prepared before the start of the study (Annexure II).

The patient and their family member or caregiver were asked to record a good-quality video of the patient’s complete inhaler technique at home in which we could see the patient taking the inhaler device with sufficient clarity to make a decision over a period of 4 weeks on specific days. The recorded video was shared with an independent inhaler technique evaluator through WhatsApp. The day of the clinic visit was considered as Day “0”. Videos recorded on Days 1, 7, 14, and 28 were assessed for inhaler use. The videos sent by the subjects were evaluated within 24–48 h. The independent evaluator, who was an experienced respiratory therapist, then scored the steps of the inhaler device used by the patient in their home environment.

### Security of the mobile device

The independent evaluator’s smartphone with videos received was secured with necessary security measures. The mobile device used by the evaluator (rater) in the study was locked with a password and fingerprint lock. The device location feature of the mobile device was activated to remotely erase data in case of loss of the device. An antivirus software was installed, and Bluetooth features were disabled. The mobile device was labeled and was exclusively used by the evaluator only to check videos. It was kept in a secure cabinet, which was always locked when not in use. All other applications including social media excluding WhatsApp were disabled or uninstalled and were not used on the mobile device. The government of India has allowed the use of WhatsApp as a tool for telemedicine in patient care.

### Scoring of inhaler technique

We created a separate score sheet to assess the efficiency of inhalation (Annexure II) for each inhaler device. A score was allotted for each step based on the criticality of each step so that the total score of each device came to 10 points. Based on the scores, we classified patients into 3 categories—effective inhalation (obtained score of 9.1–10), partially effective (obtained score of 4.1–9), and ineffective inhalation (obtained score ≤ 4). This standardized scoring sheet for the inhaler device was used at the beginning of the training of the patient and for scoring the steps in the video of the patient. Patients failing to perform >70% of the steps correctly on Day 1 video were excluded from the study as they were sent back to the physician for re-training immediately to prevent worsening of their disease status. The primary outcome was the total number of correct steps done in the technique at the end of 4 weeks. The secondary outcome looked at the most common inhaler errors made by patients. Score sheets of the inhaler technique of the patients completing the study were utilized for statistical analysis.

### Ethics approval

The study protocol was approved by the institutional ethics committee of Chest Research Foundation, Pune. Written informed consent was obtained from all the study subjects. The study was carried out in accordance with relevant guidelines and regulations.

### Statistical analysis

Repeated measures of analysis of variance were used to compare the mean score. We reported the mean step score range in 95% CI. Values <0.05 were taken statistically as significant. The analysis was conducted using the SPSS Ver. 22 and R Ver 3.4 softwares.

### Reporting summary

Further information on research design is available in the Nature Research Reporting Summary linked to this article.

## Supplementary information

Supplementary Information

Reporting Summary

## Data Availability

All the relevant data are included in the paper.

## References

[1] Barbara S, Kritikos V, Anticevich SB (2017). Inhaler technique: does age matter? A systematic review. Eur. Respir. Rev..

[2] Melzer AC (2017). Patient characteristics associated with poor inhaler technique among a cohort of patients with COPD. Respir. Med..

[3] Zainudin BMZ, Biddiscombe M, Tolfree SEJ, Short M, Spiro SG (1990). Comparison of bronchodilator responses and deposition patterns of salbutamol inhaled from a pressurised metered dose inhaler, as a dry powder, and as a nebulised solution. Thorax.

[4] Cochrane MG, Bala MV, Downs KE, Mauskopf J, Ben-Joseph RH (2000). Inhaled corticosteroids for asthma therapy: patient compliance, devices, and inhalation technique. Chest.

[5] Price DB (2017). Inhaler errors in the CRITIKAL study: type, frequency, and association with asthma outcomes. J. Allergy Clin. Immunol. Pract..

[6] Lewis A (2016). The economic burden of asthma and chronic obstructive pulmonary disease and the impact of poor inhalation technique with commonly prescribed dry powder inhalers in three European countries. BMC Health Serv. Res..

[7] J. P. M, M.W. N (2009). Oral inhalation therapy: Meeting the challenge of developing more patient-appropriate devices. Expert Rev. Med. Devices.

[8] Sanchis, J., Gich, I. & Pedersen, S. Systematic review of errors in inhaler use: has patient technique improved over time? *Chest.*10.1016/j.chest.2016.03.041 (2016). 10.1016/j.chest.2016.03.041 27060726

[9] Melani AS (2011). Inhaler mishandling remains common in real life and is associated with reduced disease control. Respir. Med..

[10] Basheti IA, Obeidat NM, Reddel HK (2017). Effect of novel inhaler technique reminder labels on the retention of inhaler technique skills in asthma: a single-blind randomized controlled trial. npj Prim. Care Respir. Med..

[11] Sanchis J, Corrigan C, Levy ML, Viejo JL (2013). Inhaler devices-from theory to practice. Respir. Med..

[12] Aydemir Y (2015). Assessment of the factors affecting the failure to use inhaler devices before and after training. Respir. Med..

[13] De Blaquiere, P., Christensen, D. B., Carter, W. B. & Martin, T. R. Use and misuse of metered-dose inhalers by patients with chronic lung disease: a controlled, randomized trial of two instruction methods. *Am. Rev. Respir. Dis*. 10.1164/ajrccm/140.4.910 (1989). 10.1164/ajrccm/140.4.910 2679269

[14] Shim, C. & Williams, M. H. The adequacy of inhalation of aerosol from canister nebulizers. *Am. J. Med*. 10.1016/S0002-9343(80)80016-7 (1980). 10.1016/s0002-9343(80)80016-7 7446554

[15] Global Initiative for Chronic Obstructive. 2018 Global Strategy for Prevention, Diagnosis and Management of COPD. *Glob. Obstr. Lung Dis*. 10.1097/00008483-200207000-00004 (2018).

[16] Global Strategy for Asthma Management and Prevention. 2018 GINA Report. https://ginasthma.org/wp-content/uploads/2019/01/2018-GINA.pdf. Accessed on 04 Oct 2020.

[17] Global Initiative for Asthma. Global strategy for asthma management and prevention (2019 report). https://ginasthma.org/wp-content/uploads/2019/06/GINA-2019-main-report-June-2019-wms.pdf (2019). Accessed 04 Oct 2020.

[18] Singh, M. WhatsApp reaches 400 million users in India, its biggest market. https://techcrunch.com/2019/07/26/whatsapp-india-users-400-million/ (2019).

[19] Ryan D, Cobern W, Wheeler J, Price D, Tarassenko L (2005). Mobile phone technology in the management of asthma. J. Telemed. Telecare.

[20] Sulaiman I (2018). A randomised clinical trial of feedback on inhaler adherence and technique in patients with severe uncontrolled asthma. Eur. Respir. J..

[21] Toumas-Shehata M, Price D, Amin Basheti I, Bosnic-Anticevich S (2014). Exploring the role of quantitative feedback in inhaler technique education: a cluster-randomised, two-arm, parallel-group, repeated-measures study. npj Prim. Care Respir. Med ..

[22] NICE. Chronic obstructive pulmonary disease in adults over 16. https://www.nice.org.uk/guidance/ng115 (2017).

[23] National Institute for Health and Care Excellence. Asthma: diagnosis, monitoring and chronic asthma management chronic asthma management. NICE guidelines. https://www.nice.org.uk/guidance/ng80 (2017).

[24] Rootmensen GN (2007). Reliability in the assessment of videotaped inhalation technique. J. Aerosol Med..

[25] Verver S, Poelman M, Bögels A, Chisholm SL, Dekker FW (1996). Effects of instruction by practice assistants on inhaler technique and respiratory symptoms of patients. A controlled randomized videotaped intervention study. Fam. Pract..

[26] Shields MD, ALQahtani F, Rivey MP, McElnay JC (2018). Mobile direct observation of therapy (MDOT) - a rapid systematic review and pilot study in children with asthma. PLoS ONE.

[27] DS C, CW C, SJ S, CN M, FJ M (2003). An Internet-based store-and-forward video home telehealth system for improving asthma outcomes in children. Am. J. Health Syst. Pharm..

[28] Van Der Palen J, Klein JJ, Kerkhoff AHM, Van Herwaarden CLA (1995). Evaluation of the effectiveness of four different inhalers in patients with chronic obstructive pulmonary disease. Thorax.

[29] Pothirat C (2015). Evaluating inhaler use technique in COPD patients. Int. J. Chron. Obstruct. Pulmon. Dis..

[30] Gregoriano C (2018). Use and inhalation technique of inhaled medication in patients with asthma and COPD: data from a randomized controlled trial. Respir. Res..

[31] Gray SL, Nance AC, Williams DM, Pulliam CC (1994). Assessment of interrater and intrarater reliability in the evaluation of metered dose inhaler technique. Chest.

[32] Liu, C. Worldwide internet and mobile users. eMarketer’s updated estimates for 2015–2020. https://www.emarketer.com/Report/Worldwide-Internet-Mobile-Users-eMarketers-Updated-Estimates-Forecast-20152020/2001897 (2015).

[33] Koch H (2017). WhatsApp Messenger as an adjunctive tool for telemedicine: an overview. Interact. J. Med. Res..

[34] Morita PP (2019). A patient-centered mobile health system that supports asthma self-management (breathe): design, development, and utilization. JMIR mHealth uHealth.

[35] Lopez Jove O (2017). Information and communication technology use in asthmatic patients: a cross-sectional study in Latin America. ERJ Open Res..

[36] Pritchard JN, Nicholls C (2014). Emerging technologies for electronic monitoring of adherence, inhaler competence, and true adherence. J. Aerosol Med. Pulm. Drug Deliv..

[37] Goodyer L, Savage I, Dikmen Z (2006). Inhaler technique in Turkish people with poor English: a case of information discrimination?. Pharm. World Sci..

