# Supplementary material for: Monitoring of inhaler use at home with a smartphone video application in a pilot study
Source: NPJ Prim Care Respir Med. 2020 Oct 16;30:46. doi: 10.1038/s41533-020-00203-x (PMC7567806; doi:10.1038/s41533-020-00203-x)
Supplement: Supplementary file 1 — Supplementary Information [file 41533_2020_203_MOESM1_ESM.pdf]

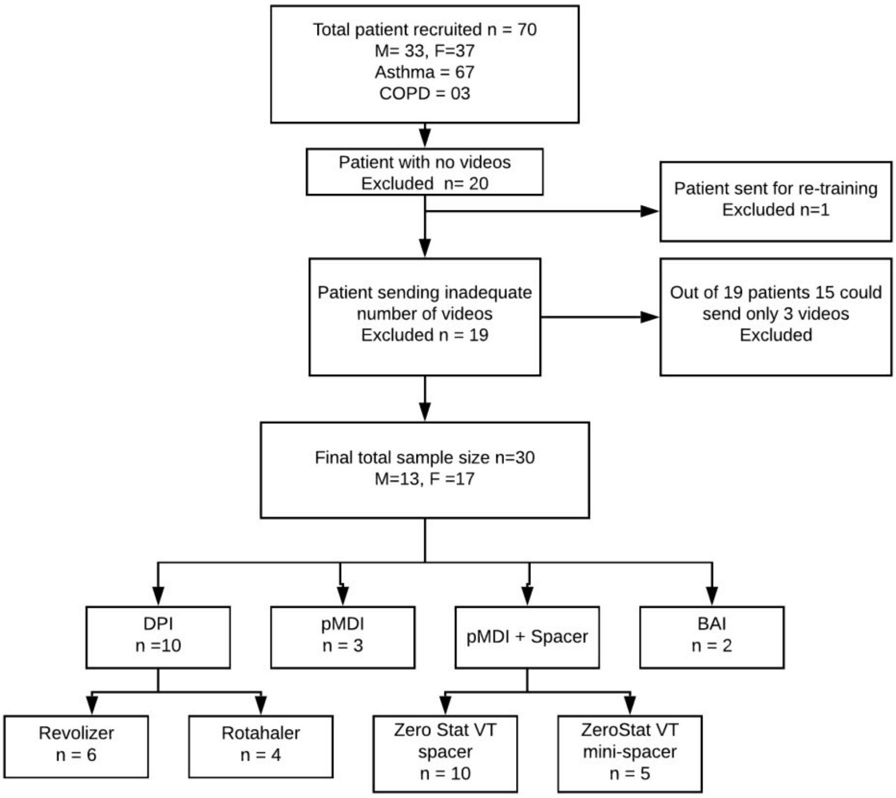

Figure 1 – Inhaler devices used in the study. M=Male, F=Female, DPI=Dry powder inhaler, MDI=pressurized metered dose inhaler, BAI= Breath actuated inhaler

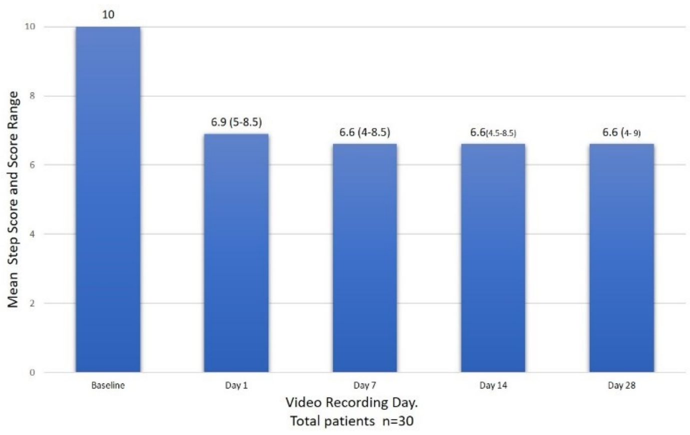

a)

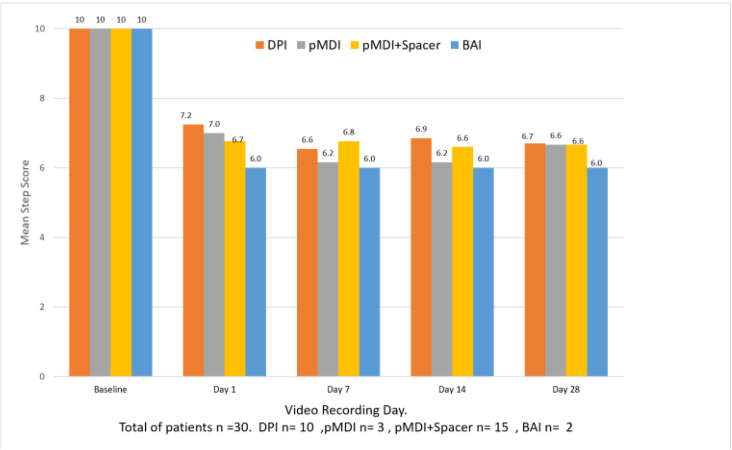

b)

Figure 2 – Mean step score with range a) All devices**.** b) Steps performed correctly per device. Range for DPI (Day 1 video 6.5-8, Day 7 video 5-8.5, Day 14 video 5.5-8, Day 28 video 4-8). Range for pMDI (Day 1 video 6-8, Day 7 video 4.5-8, Day 14 video 4.5-8, Day 28 video 6-8). Range for pMDI+Spacer (Day 1 video 5-8.5, Day 7 video 4-8.5, Day 14 video 5-8.5, Day 28 video 4-9). Range BAI (Day 1 video 5-7, Day 7 video 4-8, Day 14 video 5-7, Day 28 video 5-7).

Table 1a Most common errors of DPI

DPI (n=10)				
Steps	Day1 video	Day 07 video	Day 14 video	Day 28 video
Holds device inserts capsule	(n= 0) 0%	(n= 1) 10%	(n= 0) 0%	(n= 1) 10%
Twists/closes device	(n= 0) 0%	(n= 1) 10%	(n= 0) 0%	(n= 1) 10%
Gentle deep exhalation	(n= 3) 30%	(n= 6) 60%	(n= 5) 50%	(n= 5) 50%
Mouthpiece between teeth and seals	(n= 1) 10%	(n= 1) 10%	(n= 1) 10%	(n= 1) 10%
Inhales quickly and deeply	(n= 5) 50%	(n= 7) 70%	(n= 7) 70%	(n= 7) 70%
10 second breath hold	(n= 0) 0%	(n= 2) 20%	(n= 2) 20%	(n= 0) 0%
Exhales through nose	(n= 4) 40%	(n= 5) 50%	(n= 5) 50%	(n= 5) 50%
Gargles	(n= 8) 80%	(n= 8) 80%	(n= 8) 80%	(n= 8) 80%
Error realisation	(n= 10) 100%	(n= 10) 100%	(n= 10) 100%	(n= 10) 100%

Table 1b Most common errors of pMDI.

pMDI (n=3)				
Steps	Day1 video	Day 07 video	Day 14 video	Day 28 video
Cap open and shakes inhaler	(n= 0) 0%	(n= 0) 0%	(n= 0) 0%	(n= 0) 0%
Gentle deep exhalation	(n= 0) 0%	(n= 0) 0%	(n= 0) 0%	(n= 0) 0%
Mouthpiece between teeth and seals	(n= 0) 0%	(n= 0) 0%	(n= 1) 33%	(n= 0) 0%
Actuation and slow deep inhalation	(n= 3)100%	(n= 3)100%	(n= 3)100%	(n= 3)100%
10 second breath hold	(n= 1) 33%	(n= 2) 67%	(n= 2) 67%	(n= 2) 67%
Exhales through nose	(n= 0) 0%	(n= 1) 33%	(n= 1) 33%	(n= 0) 0%
Gargles	(n= 0) 0%	(n= 2) 67%	(n= 2) 67%	(n= 2) 67%
Error realisation	(n= 3)100%	(n= 3)100%	(n= 3)100%	(n= 3)100%

Table 1c Most common errors in pMDI+Spacer.

pMDI with Spacer (n= 15)				
Steps	Day1 video	Day 07 video	Day 14 video	Day 28 video
Open cap and shake inhaler	(n= 3) 20%	(n=4) 27%	(n=4) 27%	(n=1) 7%
Assemble spacer	(n=0) 0%	(n=1) 7%	(n=0) 0%	(n=1) 7%
Attach correctly to the spacer	(n=0) 0%	(n=1) 7%	(n=1) 7%	(n=0) 0%
Gentle deep exhalation	(n=7) 47%	(n=6) 40%	(n=12) 80%	(n=9) 60%
Hold mouthpiece between teeth and seal lips	(n=1) 7%	(n=1) 7%	(n=1) 7%	(n=0) 0%
Actuation and slow deep inhalation	(n=13) 87%	(n=11) 73%	(n=11) 73%	(n=13) 87%
10 second breath hold	(n=0) 0%	(n=4) 27%	(n=3) 20%	(n=14) 40%
Exhales through nose	(n=7) 47%	(n=5) 33%	(n=5) 33%	(n=4) 27%
Gargles	(n=12) 80%	(n=12) 80%	(n=12) 80%	(n=10) 67%
Error realisation	(n=15) 100%	(n=15) 100%	(n=15) 100%	(n=15) 100%

Table 1d Most common errors in BAI.

BAI (n=2)				
Steps	Day 1 video	Day 7 video	Day 14 video	Day 28 video
Cap open and shakes inhaler	(n=1) 50.00%	(n= 0)0.00%	(n=1) 50.00%	(n=1) 50.00%
Gentle deep exhalation	(n=0) 0.00%	(n=0) 0.00%	(n=0) 0.00%	(n=0) 0.00%
Mouthpiece between teeth and seals	(n=0) 0.00%	(n=0) 0.00%	(n=0) 0.00%	(n=0) 0.00%
Slow deep inhalation	(n=1) 50.00%	(n=1) 50.00%	(n=1) 50.00%	(n=1) 50.00%
10 second breath hold	(n=0) 0.00%	(n=1) 50.00%	(n=0) 0.00%	(n=0) 0.00%
Exhales through nose	(n=2) 100.00%	(n=2) 100.00%	(n=2) 100.00%	(n=2) 100.00%
Gargles	(n=2) 100.00%	(n=1) 50.00%	(n=2) 100.00%	(n=2) 100.00%
Error realisation	(n=2) 100.00%	(n=2) 100.00%	(n=2) 100.00%	(n=2) 100.00%

Table 2 Inhalation effectiveness score

	Day 1 video	Day 7 video	Day 14 video	Day 28 video
Effectiveness Score	(n) Percentages	(n) Percentages	(n) Percentages	(n) Percentages
Ineffective inhalation (%)<br>(4 and below)	(n=0) 0%	(n=2) 7%	(n=0) 0%	(n=2) 7%
Partially effective inhalation (%)<br>(4.1 to 9)	(n=30) 100%	(n=28) 93%	(n=30) 100%	(n=28) 93%
Effective (%)<br>(9.1 to 10)	(n=0) 0%	(n=0) 0%	(n=0) 0%	(n=0) 0%

Table3 Other observations during video monitoring.

• The patient with pMDI actuated the inhaler correctly but inhaled through the nose instead of the mouth. All the medicine escaped from the device
• The patient with pMDI and spacer actuated the inhaler in air and then attached it to the spacer for inhalation.
• The patient closed mouth during breath-hold but continued breathing through the nose.
• Patient drinking water instead of gargling
• Actuating pMDI multiple times in spacer before inhaling

Annexure I

Protocol for inhaler device training

After the diagnosis of bronchial asthma or COPD was made, the patient was explained about the diagnosis and the management plan. All investigators followed steps given below in training inhaler technique to the patient which was selected as per existing guidelines and ability of the patient to use it correctly.

1. Steps in inhaler training
    a. First, a single Inhaler device was chosen by the pulmonologist.
    b. Various parts of the inhaler device were explained personally by the pulmonologist
    c. Video of inhaler technique available from the manufacturer was shown to the patient
    d. The steps in the use of the inhaler device were explained again to the patient.
    e. The patient was asked to demonstrate how he/she would take the inhaler
    f. Any mistakes done in the first attempt were promptly corrected and explained by the pulmonologist.
    g. The patient was given the trial of the selected inhaler device to the satisfaction of the pulmonologist and achieving 100 percent score as per the scoring sheet of the device
2. The inhaler device was changed in case the patient failed to perform correct steps on the first device and then the same procedure as in Serial no. 1 was used.
3. The patient's relative or caregiver was explained how to record a video using a smartphone so that all steps of the inhaler can be seen clearly and how to send the video to the independent evaluator's WhatsApp number.

Annexure II

score sheets

Dry Powdered Inhaler Scoresheet

Device: Rotahaler / Revolizer

Step Number	Details of step of using DPI	Score for Done correctly	Score for error Done	Score for Not Done
1.	The patient holds the device correctly and inserts the capsule into the designated hole	1	0.5	0
2.	Patient twists sharply forwards and backwards (Rotahaler) / Closes the device for the needles to pierce (Revolizer)	1	0.5	0
3.	Patient breathes out gently and deeply (exhales completely)	1	0.5	0
4.	Places the mouthpiece between teeth and seals with lips	1	0.5	0
5.	The patient inhales quickly and deeply through the mouth	1.5	1	0
6.	Holds breath for 10 seconds	1.5	1	0
7.	Exhales slowly through the nose	1	0	0
8.	Gargles with water	1	0.5	0
9.	Error Realization: Makes new attempt to correct technique if not done or done incorrectly in step 1 to 8	1	0.5	0
	**Total Score**	10		

<u>Pressurized Metered Dose Inhaler Scoresheet</u>

**Step Number**	**Details of step of using pMDI**	**Score for Done correctly**	**Score for error Done incorrectly (Positive score for error)**	**Score for Not Done**
1.	The patient removes cap and shakes the inhaler	1	0.5	0
2.	Patient breathes out gently and deeply (exhales completely)	1	0.5	0
3.	Places the mouthpiece between teeth and seals with lips	1	0.5	0
4.	Actuates the canister at the beginning of inspiration and inhales slowly and deeply	2	1	0
5.	Holds breath for 10 seconds	2	1	0
6.	Exhales slowly through the nose	1	0.5	0
7.	Gargles with water	1	0.5	0
8.	Error Realization: Makes new attempt to correct technique if not done or done incorrectly in step 1 to 8	1	0.5	0
	**Total Score**	10		

Pressurized Metered Dose Inhaler with spacer Scoresheet

Step Number	Details of step of using pMDI + spacer	Score for Done correctly	Score for error Done incorrectly	Score for Not Done
1.	The patient removes cap and shakes the inhaler	1	0.5	0
2.	Patient assembles the spacer	0.5	0	0
3.	Attaches the inhaler to the spacer correctly	0.5	0	0
4.	Patient breathes out gently and deeply (exhales completely)	1	0.5	0
5.	Places the spacer mouthpiece between teeth and seals with lips	1.5	1	0
6.	Actuates the canister at the beginning of inspiration and inhales slowly and deeply	1.5	1	0
7.	Holds breath for 10 seconds	1	1	0
8.	Exhales slowly through the nose	1	0.5	0
9.	Gargles with water	1	0.5	0
10.	Error Realization: Makes new attempt to correct technique if not done or done incorrectly in step 1 to 10	1	0.5	0
	**Total Score**	10		

Signature of Evaluator and date:
